# Return to climbing after musculoskeletal injury: a scoping review protocol of rehabilitation content, outcome measures and return to sport criteria in climbers

**DOI:** 10.1136/bmjsem-2025-003156

**Published:** 2026-02-20

**Authors:** Uzo Ehiogu, Georgia Wells, Gareth Jones, Matthew Buckthorpe, Stephen Patterson

**Affiliations:** 1 Collage of Medicine and Health, University of Birmingham, Birmingham, UK; 2 Education and Training Department, Royal Orthopaedic Hospital NHS Foundation Trust, Birmingham, UK; 3 School of Health, Leeds Beckett University, Leeds, UK; 4 Education and Research Department, FIFA Medical Centre of Excellence, Bologna, Italy; 5 St. Mary’s University, London, UK; 6 Saint Mary’s University Twickenham, Twickenham, UK

**Keywords:** Rock climbing, Sport climbing, Sports rehabilitation programs, Exercise rehabilitation

## Abstract

Climbing is an Olympic sport featuring three disciplines: lead climbing, speed climbing and bouldering. The injury burden associated with climbing has been well documented. However, the content of rehabilitation programmes, the outcome measures and the return-to-sport (RTS) criteria after injury are sparse. This review will map the content of rehabilitation programmes, examine outcome measures to inform rehabilitation and RTS using a COSMIN-aligned approach, and identify objective and subjective criteria used for return to climbing after musculoskeletal injury (MSKI). The methodological framework of Arksey and O’Malley will be applied for this scoping review. A systematic review of four online databases and a manual search of reference lists of identified articles will be used to identify relevant papers. Given the limited empirical literature on this topic, both peer-reviewed and non-peer-reviewed sources written in English will be used. Sources reporting rehabilitation, recovery, outcome measures in injured and uninjured climbers and RTS/return to climbing criteria after MSKI will be included. All climbers (elite, professional and/or recreational) of any age and sex will be included. Statistical analysis of agreement between reviewers at each stage of the review will be undertaken. This review will inform future research on the rehabilitation content after MSKI. It will also aid in designing sports-specific testing batteries and in return-to-climbing decision-making criteria. The result of this review will be relevant to clinicians, performance staff and researchers and will be disseminated through publications and presentations.

**WHAT IS ALREADY KNOWN ABOUT THIS TOPIC**

- Although injury in climbers is extremely common, there is limited understanding of the rehabilitation content at each stage, the return-to-sport (RTS) criteria and the outcome measures.

**WHAT THIS STUDY ADDS**

- This review provides a transparent, reproducible method for mapping and cataloguing what is currently known about the rehabilitation and RTS parameters of climbers following musculoskeletal injury.

**HOW THIS STUDY MIGHT AFFECT RESEARCH, PRACTICE OR POLICY**

- The results of this review will assist clinicians, performance staff and researchers in the design of rehabilitation programmes, sports-specific testing batteries and decision-making criteria for return to climbing.

## Introduction and background

 Climbing has developed from a grassroots participation sport over the past two decades into an Olympic discipline.^1^ In 2021, it debuted at the Olympic Games, featuring the three main competition disciplines (lead climbing, speed climbing and bouldering). Climbing has also seen growth at the grassroots level in both outdoor rock climbing and indoor climbing on artificial climbing walls.^2^ The sport comprises different categories of climbing, each with its own rules, injury profile and physiological characteristics. The different types of climbing include traditional climbing, bouldering, speed climbing, ice climbing and sport or lead climbing.^3^ Traditional climbing and sport or lead climbing are undertaken outdoors on natural rock formations or indoors on artificial walls. The climber is attached to a rope and is belayed by a second climber. The climber ascends the route and either places protective equipment into the rock or ascends to prefixed anchor points, attaching the rope for protection in the event of a fall.^4^ This acts as a safeguard should the climber fall during their ascent. Bouldering involves ascending or traversing a short, predetermined route. This type of climbing typically lasts 10–40 s, using powerful gymnastic-style movements to reach the end of the route. The climber is not attached to a rope, and spotters and safety mats provide safety in the event of a fall to reduce the risk of injury.^2^ Bouldering and sport climbing are now a combined discipline at the Olympic Games.^5^ Speed climbing is an Olympic sport in its own right. The climber ascends a predefined route while racing against another climber attached to an electronic belay device. The winner is the athlete who reaches the end of the route with the fastest time. All these disciplines are accessible to climbers with disabilities.^1^ This diversity of climbing activities contributes to a variety of injury types and biomechanical and physiological demands.

Climbing from a biomechanical perspective can be characterised by its kinetic and kinematic profile. It can be operationalised into three different phases: stabilisation, preparation and displacement. The stabilisation phase is typically a static phase in which the climber maintains contact with the climbing surface to establish postural stability.^6^ The preparation phase is a transition stage between the stabilisation and displacement phases. The final phase is characterised by the displacement of the body’s centre of mass from one position on the wall to another. The upper limb is an important anatomical region to understand in relation to training, injury and return to performance. The primary interface between the wall or natural rock is the hand and upper quadrant. The upper limb, especially the hand, has a smaller surface area of bone, connective tissue and muscles compared with the lower quadrant.^7^ A smaller surface area for absorption, transfer and generation forces may increase injury risk. Force and the available surface area are directly related to the mechanical stress imposed upon biological tissues.^8^ The relatively high loads through the hand, wrist, elbow and shoulder regions may increase the risk of injury in this population.^9^

It may also affect rehabilitation, physical preparation and return-to-sport (RTS) considerations for climbers and clinicians.^10^ The injury burden associated with climbing has been well documented in the disciplines with the highest participation levels (sport climbing and bouldering).^11^ The prevalence of injury has been reported to vary between 10 and 81% regardless of the cause.^12 13^ Injuries associated with impact typically involve falls from height and contact with the ground or a climbing surface. The result is often lower limb injuries at the site of the foot and/or ankle, accounting for between 10% and 50% of injuries.^14^ Non-impact injuries associated with the catastrophic failure of contractile and non-contractile tissues during strenuous loading are suggested to account for 28%–81% of injuries.^12 13^ Upper limb injuries account for the highest prevalence of acute injury affecting the fingers, wrist, elbow and shoulder regions.^9^

Chronic overuse injuries account for between 33%–44% of injuries in climbers, often after repetitive and forceful exertions of tissues over time.^14 15^ The upper limb is the anatomical region most commonly affected by chronic overuse injuries.^16^ However, while the epidemiology of injury is well documented in the literature, rehabilitation, RTS criteria and outcome measures after injury are sparse.^17^ The content of rehabilitation at each stage, progressions, goals and interventions is poorly documented. In situations in which physiotherapy and rehabilitation have been reported, they are part of surgical treatment algorithms.^18^ However, rehabilitation is often not reported in sufficient detail, and no guidance on returning to climbing and performance is provided.

Outcome measures that could inform assessment across the whole rehabilitation pathway are poorly documented.^17^ This includes which tests exist, their reliability, responsiveness, validity, the constructs they measure and whether they can be used or adapted for rehabilitation. Many tests have been developed and validated in uninjured climbers,^19^ and these may provide foundational information for use as measurement tools in a clinical setting. Therefore, the measurement properties (validity, reliability, responsiveness) of outcome measures used in healthy climbers may provide a basis for rehabilitation outcome measures across the injury-to-performance continuum. RTS criteria after climbing injuries are poorly documented in the literature.^17^ Criteria can facilitate decision-making about when a climber is ready to return to climbing.^20^ Objective markers (finger strength, endurance and climbing grade) and subjective markers (pain, confidence and training tolerance) may provide a holistic view of a climber’s readiness to return to performance. Climbing-specific RTS rules are sparse in the literature and are often not anchored to preinjury or target climbing grades. Therefore, healthcare and performance professionals may rely on implicit judgement, leading to variability, reduced performance and increased re-injury risk.^20^ Structured RTS criteria provide a defensible framework from impairment to activity and participation outcomes that matter to climbing athletes.^17^

This scoping review will map the current evidence on rehabilitation content, outcome measures and RTS criteria following musculoskeletal injury (MSKI) in climbers. Specifically, this review aims to: (1) Systematically map and catalogue the content of rehabilitation programmes and approaches by stage used to facilitate the RTS after MSKI, (2) Examine and catalogue existing outcome measures which can be used to inform rehabilitation progression and return to performance. This will include their reliability, validity, responsiveness and feasibility in a clinical environment, along with details of standard operating procedures (SOPs), using a COSMIN-aligned approach and (3) To identify and map the objective and subjective criteria used for return to climbing after MSKI.

### Objectives

The objectives of this review are to:

1. To map and report the content of upper limb rehabilitation programmes used for injured climbers by stage.
2. To catalogue outcome measures and their reliability, validity, responsiveness and feasibility, which can be used to inform the progression of injured climbers.
3. To identify the RTS and return to play criteria used by performance and healthcare professionals.
4. To outline gaps in the literature to inform future research priorities.

## Methods

### Protocol and registration

This protocol will be conducted in accordance with the Joanna Briggs Institute (JBI) evidence synthesis guidelines,^21^ which requires the following steps: (a) Identify the research question or topic, (b) Identify relevant studies, (c) Study selection, (d) Charting the data and (e) Collating, summarising and reporting the results. The review will also be reported using the Preferred Reporting Items for Systematic Reviews and Meta-Analyses extension for Scoping Reviews (PRISMA-ScR).^22^ The precision of this review has been further enhanced through the use of an expanded population, concept, context and tool paradigm to focus the title, aims and objectives of this review^21^ (table 1). This approach ensures a robust and comprehensive search strategy, transparency, rigorous reporting, synthesis and presentation of the findings. This protocol will be available and prospectively registered on the Open Science Framework.

**Table 1:** Expanded population, concept, context and tool paradigm

Population	Concept	Context	Tools
Boulders	Rehabilitation and recovery	Musculoskeletal injury (finger, hand, wrist, elbow, shoulder, lower limb)	Objective tests Subjective tools Composite protocols Measurement details
Lead climbers	Return to sport/return to climbing	In any setting	
Traditional climbers	Outcome measures (impairment, activity and participation)	Any study design – Including case series, case reports, protocols, expert opinion, consensus statements, narrative reviews, clinical commentary and measurement property papers	
Speed climbers	Rehabilitation and return to sport decision/ readiness criteria (subjective and objective criteria, progression criteria, exposure criteria, physical readiness, psychological readiness)		
Ice climbers			
All ages/ indoors and outdoors			

### Eligibility criteria

The following criteria will be applied to the search for this scoping review because preliminary work indicates sparse empirical literature relevant to its objectives. Therefore, peer-reviewed and non-peer-reviewed literature, including systematic reviews, cohort studies, case series, case studies, clinical practice guidelines and expert opinion papers/articles reporting rehabilitation and recovery after MSK injury. In addition, outcome measures aligned with the International Classification of Functioning, Disability and Health (ICF) at the impairment, activity and participation levels in injured and uninjured climbers will be included. This search approach will also be applied to RTS/return-to-climbing criteria after MSK injury. Eligible studies must be written in English and include climbers (professional and/or recreational) of any level, age and sex as the target of intervention or its objectives. Databases will be searched from 2000 or the date of inception, whichever is earlier. Studies focusing on concussion, facial or eye injuries will be excluded. Epidemiological studies without reference to return to climbing, RTS, rehabilitation or outcomes will be excluded. Website blogs and social media sites will not be considered unless associated with a peer-reviewed periodical (eg, British Journal of Sports Medicine).

### Information sources and search strategy

To ensure the search strategy is as comprehensive as possible, a three-step approach will be used, as recommended by JBI.^21^ In the first step, the lead author, working with a faculty librarian, will conduct an initial limited search of two databases (PubMed and CINAHL) using the following search strings:

#### Rehabilitation content

“climbing” OR “bouldering” OR “sport climbing” OR “rock climbing” OR “lead climbing” OR “trad climbing” OR “traditional climbing” OR “ice climbing” AND “rehabilitation” OR “physical therapy” OR “physiotherapy” OR “exercise therapy” OR “return to sport” OR “return to play” OR “return to climbing” OR “return to performance” AND “Injury” OR “strain” OR “tear” OR “pulley injury” OR “A2” OR “tendon” OR “tendinopathy” OR “tendinitis” OR “soft tissue” OR “upper limb” OR “shoulder” OR “elbow” OR “wrist” OR “hand” OR “finger” OR “knee” OR “ankle” OR “heel hook” OR “ACL” OR “anterior cruciate ligament”.

#### Outcome measures

“climbing” OR “bouldering” OR “sport climbing” OR “rock climbing” OR “lead climbing” OR “trad climbing” OR “traditional climbing” OR “ice climbing” AND “readiness” OR “criteria” OR “protocol” OR “outcome” OR “measure” OR “outcome measures” OR “patient reported outcome measures” OR “battery” OR “Protocol” AND “grip” OR “grip strength” OR “finger strength” OR “dead hang” OR “hang board” OR “campus board” OR “pull ups” OR “isometric mid-thigh pull” OR “dynamometer” OR “contact strength” OR “handgrip”.

#### Return to sport/climbing decision-making criteria

“climbing” OR “bouldering” OR “sport climbing” OR “rock climbing” OR “lead climbing” OR “trad climbing” OR “traditional climbing” OR “ice climbing” AND “rehabilitation” OR “physical therapy” OR “physiotherapy” OR “exercise therapy” OR “return to sport” OR “return to play” OR “return to climbing” OR “return to performance” AND “readiness” OR “criteria” OR “protocol” OR “outcome” OR “measure” OR “outcome measures” OR “patient reported outcome measures” OR “battery” OR “Protocol”.

After this initial search, the titles and abstracts of the retrieved papers will be analysed. A second search using all identified index terms and keywords will be conducted across all databases, including Embase, Cochrane and Medline. The reference list of all identified articles will be searched for additional literature. The databases have been selected based on their relevance to research topics in health, sport medicine and clinimetrics, and the search strings will be adapted as appropriate for each database. The third stage will identify the reference lists of all the literature selected as full text or included in the review. The authors intend to contact the authors of primary studies or reviews for further information if required.

### Screening and selection

Following the search, all identified records will be collected and uploaded to Rayyan (Qatar Computing Research Institute), and duplicates will be removed. After pilot testing of the software, the titles and abstracts will be screened by two reviewers (UE and GW) for eligibility in the review. Potentially relevant studies will be retrieved in full and their citation details imported into the Rayyan web-based tool. The full texts of selected citations will be assessed in detail by two reviewers against the inclusion and exclusion criteria. Any disagreements between the reviewers at each stage of the selection process will be adjudicated by a third reviewer (GJ). The reasons for inclusion and exclusion will be recorded and reported in the scoping review. The authors of papers that require missing or additional data will be contacted. If access to missing data is not available, then these papers will be excluded. The results of the search strategy will be reported in full in the final review and presented in a PRISMA-ScR flow diagram.

### Statistical analysis

Inter-rater agreement between the reviewers will be assessed using Cohen’s kappa (k) to quantify agreement. A χ^2^ test will be used to assess the statistical significance of agreement between reviewers.^23^ This will be applied to stage two and stage three of the search strategy using SPSS V.29.020(20) (IBMY).

### Data charting (extraction)

Data charting, collection and extraction for the review will follow a systematic, transparent process to ensure the capture of all relevant information.^21^ The data will be captured in a table for easy comparison in Excel 2021, based on the JBI manual, into three key domains of the following:

- Rehabilitation content at various stages.
- Outcome measures.
- RTS/climbing decision-making criteria.

### Data items

The standardised data extraction form will capture key biographical and demographic data, including: author, year of publication, sample size, research design, sex, age, type of climber (eg, Lead climber), and standard (elite vs recreational). Then, specific information will be captured from each domain (table 2) and recorded in the standardised data extraction form.^24^ A COSMIN-aligned approach will be used to extract and appraise measurement properties (eg, reliability, validity, responsiveness) of outcome measures identified in the review. This will ensure consistent reporting and enable mapping of evidence gaps relevant to climbing rehabilitation and RTS criteria. The data will be extracted by two reviewers (UE and GW) to minimise bias, with discrepancies resolved by discussion with (GJ) the third reviewer.

**Table 2:** Specific information captured from each domain

Rehabilitation content at various stages		Outcome measures		Return to sport/climbing decision-making criteria
Injury region and rehabilitation stage	Injury type/Region Acute, Chronic, Intermediate and Advanced RTP Stages	Outcomes	ICF Domain (eg, Impairment, activity, participation), Construct Assessed (eg, finger flexor strength/endurance), Outcome name (eg, finger dynamometry)	Context (Rehab/Return-to-Training/Return-to-Performance), RTS Definition Used (Author’s Description), Decision-Making Model (eg, phased, criteria-based, time-based, hybrid), ICF Domain (Impairment/Activity/Participation)
Rehab content (themes) and Type of Intervention	Key Intervention Themes (eg, tendon-isometrics, “progressive hang board training, graded exposure, manual therapy, power training, climbing skill/movement training) One line description Example quote	Reliability and validity	Test-retest (statistic/ICC/r), Intra-rater, Inter-rater, Internal consistency (Cronbach’s alpha), Measurement error (SEM/CoV/LoA) Content Validity, Face Validity, Construct Validity, Criterion Validity, Structural Validity, Responsiveness, Anchor-Based Responsiveness, Minimal Detectable Change, Minimal Clinically Important Difference, RTS Threshold, Anchor Used (eg, return to redpoint grade), ceiling/Floor Effects (%)	
Outcome measures mentioned/used	ICF Domain (eg, pain, functional outcome measures, finger strength endurance) Reliability as mentioned Construct Assessed (eg, finger flexor strength/endurance), Outcome name (eg, finger dynamometry)	Test SOP details	Outcome description (eg, how the test is performed), Equipment set up (eg, edge depth, grip type), Load parameters (% Body weight, reps, time), kg or allometric scaling	Primary Construct (eg, strength, pain, function, confidence), Objective Criteria Reported (Yes/No), Objective Tests Used (eg, grip, finger strength, CMJ, endurance), Objective Threshold (value/units/% symmetry), Threshold Type (Guarded/Grade-Anchored/Consensus/Statistical), Anchor Used (eg, preinjury grade/return event/functional test), Measurement Property Evidence (Reliability/Validity/Responsiveness), Subjective Criteria Reported (Yes/No), Subjective Measures (eg, pain, fear, confidence, readiness), Tool Used (eg, VAS, PRRS, GROC, confidence scale), Subjective Threshold/Cut-off (if any), Anchor (eg, self-rated readiness/symptom-free), Pain Criteria (Yes/No; specify scale or limit)
RTS/RTP criteria, if mentioned:	Same data charting as Return to sport/climbing criteria	Feasibility /safety notes		Functional Criteria (eg, ability to perform movement/climb type), Strength/Load Criteria (eg, % of contralateral/% preinjury), Psychological Readiness (Yes/No), Fatigue/Endurance Criteria (Yes/No), Movement Quality or Control Criteria (Yes/No), Sport-Specific Task Criteria (eg, wall, grade, movement), Time-Based Criteria (weeks/phases/post-op milestones), Clinical Judgement/Consensus Used (Yes/No), Multidisciplinary Involvement (Yes/No; specify roles)
Feasibility/safety notes	Equipment required, stop rules, adverse events and Test completion time		Safety/contraindications (eg, Stop rules), Adverse events Testing time (mins), Resources required, Acceptability to clinician/patient	Environmental/Equipment Considerations (eg, wall type, hold type)
Key results/findings	Author reported limitations, Reviewer Notes/Comments	Key results/findings	Author reported limitations, Reviewer Notes/Comments	Key results/Findings, Level of Evidence (eg, expert opinion, consensus, empirical study), Reported Outcome after RTS (eg, recurrence, re-injury, performance)
				Author reported limitations, Reviewer Notes/Comments

CMJ, counter movement jump; ICC, intraclass correlation coefficient; ICF, International Classification of Functioning, Disability and Health; RTP, return to play; RTS, return to sport; SOP, standard operating procedure.

### Synthesis plan

A PRISMA flow diagram will be used to document the selection process, including database searching, recording, screening and the final selection of studies for inclusion.^22^ The selected studies will be presented in three tables and descriptively mapped by domain: rehabilitation content at various stages, outcome measures and RTS/climbing decision-making criteria. The outcome measures table will map the different measurement tools used in each paper to validity, reliability, responsiveness, feasibility and test administration. A heat map of the outcomes used will be presented, aligned with the ICF.^25^ The second and third tables will list rehabilitation content by stage and the RTS criteria factors identified in protocols and published sources. A narrative synthesis of the data will involve categorising and summarising RTS criteria, outcome measures and rehabilitation content. This will include the interventions and tools used, the populations studied, gaps in understanding and future challenges.

## Discussion

The sport of climbing is undergoing significant growth due to its transition to the Olympics and its mass participation at the grassroots level.^26^ However, because of its minority status, there is a lack of guidance for clinicians and performance staff on rehabilitation, outcome measures and decision-making criteria for RTS after MSKI.^27^ The structure of rehabilitation programmes after injury is poorly reported and not standardised. The nature of rehabilitation after climbing injuries, when reported, is a combination of heterogeneous case studies,^28^ clinical commentaries^27 29^ and expert opinion pieces.^30^ There is a need to understand what is being undertaken at each stage of a climber’s rehabilitation, which parameters are used as progression criteria between stages, and whether there is consistency in reporting.

The nature of outcome measures used to direct rehabilitation interventions is not well established. However, there is an abundance of outcome measures used in uninjured populations.^19^ It is necessary to understand their collective validity, reliability (ICC/SEM/MDC95), responsiveness to change and their feasibility in a clinical setting. Outcome measures of physical capacity, combined with an athlete’s pain tolerance during these tests in a clinical setting, may serve as a safety threshold for progression between and through stages of rehabilitation. Therefore, studies which use outcome measures to predict and document performance in uninjured climbers may provide the infrastructure (SOPs) and a starting point for clinical testing.

The decision to return to climbing is a balance between physical and psychological readiness and risk management and should therefore be evidence-based.^31^ At present, the decision criteria are not well documented and are often time-based, symptom-based or based on the empirical knowledge of the clinician and performance staff. Objective benchmarks are not well established, and it is uncertain if the criteria currently used are anchored to climbing grade. RTS criteria, which are anchored to climbing grade, are an obvious performance marker because of their close relationship with the climber’s preinjury level of performance. This type of measure is aligned with the ICF^25^ and reflects participation in important goal-oriented activities meaningful to climbers.

There is currently no example in the literature of a scoping review mapping the rehabilitation content, outcome measures and RTS criteria for musculoskeletal climbing injuries. However, there are many examples of this approach in healthcare used to identify gaps in research and clinical practice. The results of this scoping review will help develop future RTS outcome measures and research priorities for climbing rehabilitation.

### Stakeholder consultation

The Delphi panel of an ongoing study will be consulted with preliminary maps to prioritise outcome measures and criteria for future studies.^32^

## Data Availability

Data are available upon reasonable request.
